# Cost-Effectiveness of Brexucabtagene Autoleucel versus Best Supportive Care for the Treatment of Relapsed/Refractory Mantle Cell Lymphoma following Treatment with a Bruton’s Tyrosine Kinase Inhibitor in Canada

**DOI:** 10.3390/curroncol29030164

**Published:** 2022-03-17

**Authors:** Graeme Ball, Christopher Lemieux, David Cameron, Matthew D. Seftel

**Affiliations:** 1 Gilead Sciences Canada, Inc., Mississauga, ON L5N 2W3, Canada; graeme.ball@gilead.com; 2 Medicine Department, Laval University, Québec, QC G1V 0A6, Canada; christopher.lemieux.2@ulaval.ca; 3 PIVINA Consulting Inc., Mississauga, ON L4W 5B2, Canada; dcameron@pivina.com; 4 Department of Medicine, University of British Columbia, Vancouver, BC V1Y 1T3, Canada; 5 Canadian Blood Services, Vancouver, BC V6T 1V6, Canada

**Keywords:** chimeric antigen receptor T cell therapy, gene therapy, cost-effectiveness

## Abstract

For patients with Mantle Cell Lymphoma (MCL), there is no recognized standard of care for relapsed/refractory (R/R) disease after treatment with a Bruton’s tyrosine kinase inhibitor (BTKi). Brexucabtagene autoleucel (brexu-cel) represents a promising new treatment modality in MCL. We explored whether brexu-cel was cost-effective for the treatment of R/R MCL. We developed a partitioned survival mixture cure approach to model the costs and outcomes over a lifetime horizon. The clinical data were derived from the ZUMA-2 clinical trial. The costs were estimated from the publicly available Canadian databases, published oncology literature, and pan-Canadian Oncology Drug Review economic guidance reports. The health state utilities were sourced from the ibrutinib submission to the National Institute for Health and Care Excellence for R/R MCL and supplemented with values from the published oncology literature. In the base case over a lifetime horizon, brexu-cel generated an incremental 9.56 life-years and an additional 7.03 quality-adjusted life-years compared to BSC, while associated with CAD 621,933 in additional costs. The resultant incremental cost-utility ratio was CAD 88,503 per QALY gained compared with BSC. Based on this analysis, we found brexu-cel to be a cost-effective use of healthcare resources relative to BSC for treatment of adult patients with R/R MCL previously treated with a BTKi in Canada, though additional research is needed to confirm these results using longer follow-up data.

## 1. Introduction

Mantle cell lymphoma is a frequently aggressive subtype of non-Hodgkin lymphoma (NHL), estimated to account for 2–6% of newly diagnosed cases of NHL [1,2,3,4]; in Canada, there are approximately 1500 prevalent cases of MCL, and around 400 new cases diagnosed each year [5].

Patients with MCL typically present with generalized lymphadenopathy and extranodal involvement of the blood, bone marrow, and spleen [2]. Patients are often diagnosed with advanced disease (Stage III or IV), which is characterized by an aggressive clinical course and a poor prognosis [2,6,7]. The 5-year relative survival in MCL is estimated at 30–60% and is the lowest among the different NHL subtypes [6,8]. Although the response rates to frontline chemoimmunotherapy treatments are high, most patients eventually relapse and thus need additional therapy [9,10]. In addition, a proportion of patients have disease which is refractory to initial treatment [9,10,11,12,13,14]. Collectively, these patients are referred to as having relapsed or refractory (R/R) MCL. For patients who are R/R to frontline treatments, treatment options include further chemoimmunotherapy, a Bruton’s tyrosine kinase inhibitor (BTKi) or an allogeneic stem cell transplant (allo-SCT) in rare cases where possible and appropriate; the choice of treatment is influenced by the response duration to frontline therapy, comorbidities, patient age, and overall risk–benefit evaluations [2]. Most patients in Canada will receive a BTKi. However, there are no recognized standard of care options for R/R MCL patients after treatment with a BTKi. Poor outcomes have been observed in R/R MCL patients experiencing disease progression following treatment with BTKi therapy; even with subsequent treatment, the survival outcomes are poor, with a median overall survival (OS) of 1 year or less [9,10,11,12,13,14].

The absence of effective therapies for use after BTKi therapy is reflected in the current treatment guidelines, which do not include clear recommendations for treatment beyond second-line therapy [2,3,15]. There is thus a need for a novel therapeutic for patients previously treated with a BTKi. Brexucabtagene autoleucel (brexu-cel) is an autologous chimeric antigen receptor T cell (CAR T) therapy that may offer an effective treatment option for these patients. The clinical efficacy of brexu-cel was demonstrated in the phase II ZUMA-2 clinical trial. In the primary efficacy analysis, 57% of patients who received a single infusion of brexu-cel were in remission, and median OS and median PFS were not reached at a median follow-up of 12.3 months [16]. Brexu-cel received a Notice of Compliance from Health Canada in 2021 and is approved by the Food and Drug Administration (FDA); conditional authorization has also been granted by the European Medicines Agency [17,18]. In addition, brexu-cel has recently received positive reimbursement recommendations in both Canada and the UK [19,20].

Using data from the ZUMA-2 trial supplemented with the published oncology literature, this analysis explores, from a Canadian health care system perspective, the cost-effectiveness of brexu-cel versus best supportive care (BSC) for the treatment of adult patients with R/R MCL previously treated with a BTKi.

## 2. Materials and Methods

**Figure 1:**
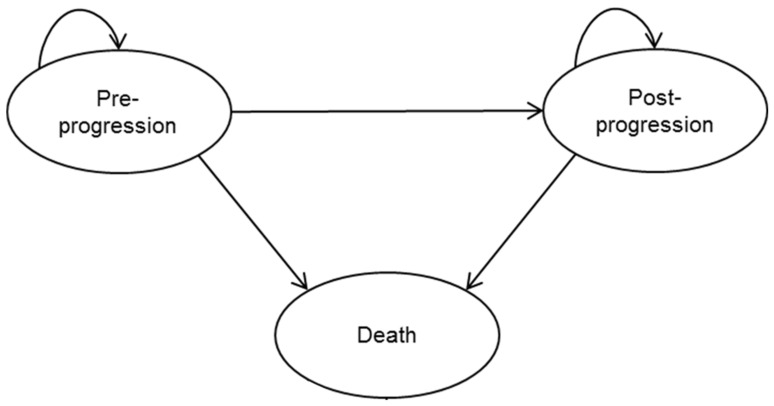
Model structure.

In order to evaluate the cost-effectiveness of brexu-cel versus BSC for adult patients with R/R MCL over a lifetime horizon from a Canadian healthcare system perspective, an economic model was developed in Microsoft Excel (Microsoft Inc., Redmond, WA, USA) using a three-health state, partitioned survival model (PSM) (Figure 1*)*. The clinical data for overall and progression-free survival (PFS) for brexu-cel were derived from the ZUMA-2 [16] study. In the absence of comparative randomized control trial (RCT) data, a historical control arm was constructed from a meta-analysis of studies that evaluated subsequent treatments in MCL patients who had been treated with a BTKi [21]. Survival estimates were extrapolated beyond the duration of the clinical studies using standard parametric fitted curves. A partitioned survival mixture cure modeling approach [22,23,24,25,26] was taken to characterize the potential to achieve long-term durable remissions with CAR T therapy in R/R MCL. Healthcare resource utilization and adverse event data were based on data from ZUMA-2 and validated through a series of structured interviews with Canadian clinical experts. The costs, reported in 2021 Canadian dollars, were derived from the publicly available Canadian cost databases and the published oncology literature. The future costs and future outcomes were discounted at 1.5% per year [27].

The primary outcome was the incremental cost-utility ratio (ICUR). Sensitivity analyses were conducted to assess the robustness of the results. A cycle length of 1 month (30.4375 days) was applied. The model inputs and data sources are summarized in Table 1.

### 2.1. Target Population

The target population for the economic analyses is defined as adult patients with relapsed or refractory mantle cell lymphoma (MCL) after two or more lines of systemic therapy, including a BTKi, in line with the intent-to-treat (ITT) patient population in the ZUMA-2 trial [16]. A total of 74 patients with confirmed R/R MCL were enrolled and underwent leukapheresis. Bridging therapy was administered at the investigator’s discretion, with the aim of ensuring the patient remained able to receive brexu-cel. Bridging therapy consisted of ibrutinib (560 mg daily) or acalabrutinib (100 mg twice daily) and/or dexamethasone (20–40 mg orally or IV daily for 1–4 days or an alternative corticosteroid) and was to be completed at least 5 days before the initiation of lymphodepletion chemotherapy. All patients received lymphodepletion chemotherapy, consisting of fludarabine (30 mg/m^2^ of body surface area [BSA] per day) and cyclophosphamide (500 mg/m^2^ of BSA per day), each given daily for 3 days (typically on Days −5 through −3 prior to receiving the CAR T cell infusion). Brexu-cel was successfully manufactured for 71 patients (96%) and administered to 68 patients (92%) and administered as a single intravenous infusion.

### 2.2. Comparators

As there is no recognized standard of care in R/R MCL, the primary comparator to brexu-cel is best supportive care (BSC), a blended comparator that includes multiple therapy options expressed as a single basket comparator with a single blended efficacy and safety profile, weighted by the proportion of patients expected to receive each therapy. In Canada, chemoimmunotherapy, such as bendamustine + rituximab, rituximab + cyclophosphamide + doxorubicin + vincristine + prednisone (R-CHOP), and R-DHAP, is most commonly used, with other therapies, including lenalidomide (+/− rituximab), bortezomib (+/− rituximab), and allogeneic transplant, being used less frequently.

### 2.3. Model Perspective

The model base case adopted a Canadian healthcare system perspective, which includes direct costs associated with the treatment and healthcare resource use of patients with R/R MCL.

### 2.4. Time Horizon, Discounting, and Cycle Length

Patients in the ZUMA-2 trial had a median age of 63.7 years, and the trial population included patients as young as 38 years old. In order to ensure that all the costs and clinical benefits related to the intervention and comparators were accounted for, the time horizon was set to 50 years. This approach should be considered appropriate, given that brexu-cel may be associated with sustained transformation of the natural history of the disease. Discount rates were set to 1.5% per year for both the costs and the benefits, in line with Canadian Agency for Drugs and Technologies in Health (CADTH) guidelines for the conduct of economic evaluations of health technologies [28].

### 2.5. Model Structure and Approach

#### 2.5.1. Partitioned Survival Model

A partitioned survival model (PSM) with three health states (pre-progression, post-progression, and death) was selected as the model structure as it is widely used in oncology modeling and previous assessments of CAR T therapy (Figure 1) [29,30]. Costs and utility values were applied to each health state. To achieve a balance between the sensitivity and the complexity of the model along with consistency with previous analyses, a cycle length of 1 month (30.44 days) was implemented.

#### 2.5.2. Partitioned Survival Mixture Cure Model

In previous CAR T clinical trials with longer-term follow-up available, a strong response dichotomy is consistently observed between CAR T treatments and their historic cohort comparators [31,32,33,34]. Mixture models allow the capture of this aspect of the data more clearly than in standard parametric models and thus better reflect the extrapolated clinical outcomes in a cost-effectiveness model. To capture the heterogeneity in population and outcomes in a partitioned survival framework, a partitioned survival mixture cure model (PS-MCM) approach was implemented which stratified the analysis according to a ‘functionally cured’ group and a ‘non-cured’ group [22]. While longer term trial evidence is required to substantiate this stratification, the `functionally cured’ patients are assumed to approach the age- and sex-adjusted mortality rates depicted in the Canadian life tables (2016–2018) [35,36]. The ‘non-cured’ patients are subject to cancer-specific hazards which are modelled and estimated using standard parametric functions.

### 2.6. Survival Estimates for Brexu-Cel

The OS and PFS Kaplan–Meier (KM) data used to inform the drug efficacy parameters were sourced from the ZUMA-2 primary efficacy analysis (data cut-off: July 24, 2019; median follow-up: 12.3 [range:7.0–32.3] months). Visual fit, statistical fit, and clinical plausibility were all considered when assessing the plausibility of different modeling and extrapolation approaches for OS and PFS, in accordance with the National Institute for Health and Care and Excellence (NICE) Decision Support Unit (DSU) 14 [37]. The initial parametric modeling of brexu-cel OS and PFS were performed by fitting the distributions recommended by CADTH for PSMs to the ZUMA-2 time-to-event data using maximum likelihood estimation [27,37]. The curves were selected based on the Akaike information criterion (AIC), Bayesian information criterion (BIC), face validity of the fit with the data and shape of the extrapolation, and clinical plausibility of the survival extrapolations. Overall survival for both treatment arms in the model was capped based on the general population mortality data taken from Canadian Life Tables 2016–2018 and adjusted for the patient characteristic profile of the ZUMA-2 ITT population [35].

### 2.7. Survival Estimates for Best Supportive Care

To model the efficacy parameters for the BSC comparator, a meta-analysis was conducted based on a systematic literature review to identify the studies that evaluated subsequent treatments in MCL patients who had been treated with BTKi [38]. The meta-analyses identified three studies for OS [9,10,13] and one study for PFS [10] that included relevant comparators, including various chemo-immunotherapies or systemic treatments, such as rituximab, bendamustine, and cytarabine (R-BAC). The parametric survival functions were fitted to reconstructed individual patient data (IPD) using the algorithm developed by Guyot et al., 2012 [39], from each relevant study. The parameters from the best fitting distribution were then pooled in a random effects meta-analysis model to provide an estimate of the absolute treatment effects in terms of OS and PFS.

### 2.8. Cost and Resource Use

All the costs presented and used in this analysis were adjusted for inflation to 2021 Canadian dollars [40]. A detailed breakdown of the costs is presented in Appendix A.

### 2.9. Drug Administration

The administration of BSC and lymphodepletion chemotherapies is assumed to include all the costs associated with the outpatient administration of chemotherapy, including the cost of physician services. Subsequent therapies were modelled using a basket approach: the costs of each comparator were weighted by the expected proportion of patients expected to receive each therapy, as per the approach taken in the cost-effectiveness model submitted to pCODR for the review of ibrutinib for MCL [5].

### 2.10. End-of-Life Costs

The cost of death was included for both treatment arms at an average cost of CAD 35,262 (2021 CAD dollars; inflated from CAD 24,015 in 2003 CAD dollars) based on a study of palliative services by patients in Ontario between 2002 and 2003 for adults who died with cancer [41].

### 2.11. Brexu-Cel Specific Treatment Costs

The costs associated with brexu-cel treatment included leukapheresis, lymphodepletion chemotherapy, bridging therapy, the acquisition cost of brexu-cel, and cell infusion and monitoring. For simplicity, all the administration costs associated with brexu-cel were assumed to be incurred in the first model cycle. Bridging therapy in ZUMA-2 was administered to patients at the discretion of the treating investigator: 36.8% of patients received bridging therapy, which included ibrutinib, acalabrutinib, or dexamethasone [42]. As acalabrutinib is not available for the treatment of MCL in Canada, it was assumed that ibrutinib and dexamethasone were the only therapies used for bridging. Aligned with the dosing observed in ZUMA-2, the model assumed that patients received 560 mg daily of ibrutinib via IV and 40 mg of oral dexamethasone daily for 4 days. The unit costs are presented in Appendix A. The administration costs were taken from the Ontario Ministry of Health and Long-Term Care (MoHLTC) Schedule of Benefits for Physician Services code G359 [43]. Lymphodepletion chemotherapy in ZUMA-2 included intravenous infusions of cyclophosphamide 500 mg/m^2^ and fludarabine 30 mg/m^2^ on the 5th, 4th, and 3rd days prior to the infusion of brexu-cel. The unit costs for cyclophosphamide and fludarabine were taken from a prior pCODR Economic Guidance Report for Ibrutinib for Mantle Cell Lymphoma [5] (Appendix A). The costs of the chemotherapy were derived after calculating the optimal combination of the different vial sizes, assuming an average body surface area (BSA) of the patients in ZUMA-2 [42]. Lymphodepletion chemotherapy was assumed to be conducted in an out-patient setting. The administration cost was taken from the Ontario Ministry of Health and Long-Term Care (MoHLTC) Schedule of Benefits for Physician Services code G359 [43]. Leukapheresis for CAR T cell manufacturing was included based on the unit costs described in a Canadian study examining the safety and cost-effectiveness of autologous stem cell transplantation in patients with multiple myeloma within a Canadian environment [44]. The cost of stem cell apheresis (not including the costs of filgrastim) was used as proxy for the cost of mononuclear cell leukapheresis. The infusion of brexu-cel and the subsequent monitoring were assumed to incur the cost of an elective hospitalization. The mean length of stay observed in the ZUMA-2 trial for patients treated with brexu-cel was 16.5 days [42]. To cost this in the model, the weighted average cost per day of an inpatient hospitalization for malignant lymphoma (Canadian Institute for Health Information Case Mix Group 615) [45] was multiplied by the mean length of stay reported in ZUMA-2. A proportion of patients from ZUMA-2 receiving brexu-cel also required monitoring within an intensive care unit (ICU) [42]. Due to a paucity of available data, this proportion was assumed to be 22% based on the use of vasopressors as a proxy for ICU admission. This proportion of patients incurred a cost per ICU day that was multiplied by the mean length of stay reported in ZUMA-2. The cost per ICU day was based on a study by Zheng et al., 2020, that reports the cost of patients with cancer that are admitted to the ICU [46].

### 2.12. BSC Specific Treatment Costs

As the BSC arm is applied as a blended comparator based on the use of a mixture of treatment regimens, the costs associated with the treatment have been weighted according to the number of patients on each treatment in the included studies. The treatments not currently publicly funded in Canada were excluded, and the treatment proportions were reweighted based on feedback from clinical experts. Table 2 presents the distribution of treatments based on proportions derived from the meta-analysis and validated by Canadian clinical experts. The treatment duration for the treatments included in the BSC arm follows the treatment regimen for each individual treatment. The proportion of patients receiving each treatment and dosage in BSC is sourced from the treatment protocols and Canadian clinical experts [47,48,49,50,51,52,53,54].

### 2.13. Drug Acquisition

The treatment dose for BSC in Canada was calculated based on body surface area (BSA). Wastage was considered in this model and dose intensity was assumed to be 100%. The costs and dosing are presented in Appendix A.

#### Health State Resource Use and Costs

Medical resource use is dependent on progression status and was therefore modelled according to health state. The subsequent therapies received in the post-progression health state were not explicitly modeled as there were no data available to enable this analysis. Healthcare resource use in each health state was estimated based on input from Canadian clinical experts. It was also assumed that patients who remain progression-free for at least 5 consecutive years are deemed to be in long-term remission. Consequently, these patients were assumed to utilize fewer medical resources (Appendix A). The unit costs of the healthcare resources were sourced from the Ontario Ministry of Health and Long-Term Care Schedule of Benefits [43,55] and the CIHI Patient Cost Estimator (PCE) [45], where applicable (Appendix A).

### 2.14. Adverse Events

Adverse events (AEs) were only applied to the brexu-cel treatment arm, and AE costs were applied as a one-off cost in the first model cycle (Appendix A). Consistent with a previously conducted study [30], the management of all AEs other than CRS was assumed to include the cost of one excess bed day; this is assumed to be captured in the reported mean length of stay of 16.5 days for ZUMA-2 patients. It was further assumed that the costs of AEs are covered in the length of stay for brexu-cel patients during cell infusion and monitoring and therefore costing each AE individually would result in double counting. The management costs for CRS were calculated using the method from the ZUMA-2 clinical study report [42].

### 2.15. Utility Values

Given the absence of published utility values for R/R MCL patients post-BTKi and the sparsity of EuroQol 5D (EQ5D) data collected in ZUMA-2, the health state utility values for both pre-progression and post-progression were sourced from the ibrutinib NICE R/R MCL submission [56] (Table 3). Based on similar demographics and the use of these values in other similar CADTH submissions, the UK estimates by Ara and Brazier (2010) [57] were used as a proxy for long-term survivors who were assumed to have a utility equal to the baseline general population utility of a 67-year-old.

## 3. Results

### 3.1. Deterministic Analysis

In the base case analysis over a lifetime horizon of 50 years, brexu-cel generated a total of 11.26 discounted life-years (LYs) compared to 1.70 LYs for BSC, leading to an incremental gain of 9.56 LYs (Table 4). In terms of QALYs, brexu-cel generated 8.34 QALYs compared to 1.31 QALYs for BSC, yielding an incremental gain of 7.03 QALYs (discounted). The total discounted costs were higher for brexu-cel (CAD 699,202) than for BSC (CAD 65,847), which led to an incremental cost difference of CAD 633,355 and an ICUR of CAD 88,503 per QALY gained.

### 3.2. Probabilistic Sensitivity Analysis

**Figure 2:**
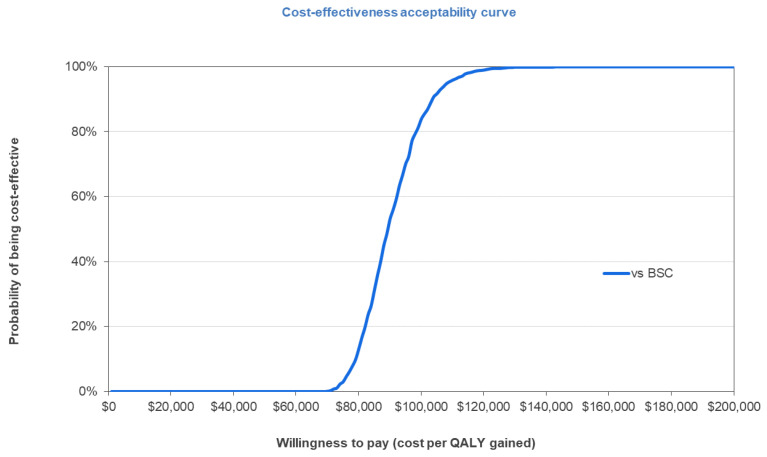
Cost-utility acceptability curve of 1000 simulations. Abbreviations: BSC, best supportive care; QALY, quality-adjusted life year.

Using 1000 Monte Carlo simulations, over a lifetime horizon of 50 years, brexu-cel was associated with higher mean total QALYs (mean incremental QALYs of 7.00) with a higher mean total cost (mean incremental cost of CAD 621,571) when compared with BSC among patients with R/R MCL who had received prior treatment with a BTKi, resulting in an ICER of CAD 88,814 per QALY gained and corroborating the results of the deterministic base case analysis. Brexu-cel was found to be cost-effective in 82% of the simulations at the commonly cited willingness-to-pay (WTP) threshold of 100,000 CAD/QALY for oncology products in Canada (Figure 2) [58].

### 3.3. Univariate Sensitivity Analysis

**Figure 3:**
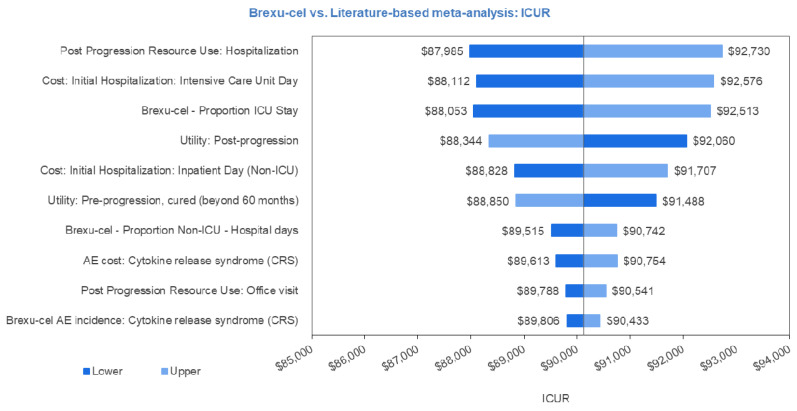
Univariate sensitivity analysis tornado plot. Abbreviations: AE, adverse event; ICU, intensive care unit.

To characterize the uncertainty associated with individual input parameter values, a univariate sensitivity analysis was conducted in which each parameter was independently adjusted to its respective lower and upper ranges. The results of the 10 most influential parameters on the ICUR are plotted onto a tornado diagram presented in Figure 3. The most influential parameters were those around the cost and length of stay of hospitalizations and the utility values of pre- and post-progression.

### 3.4. Scenario Analysis

A special interest scenario analysis was also conducted in which the post-progression survival and post-progression costs associated with treatment with brexu-cel were set exactly equal to BSC. The results of this analysis yielded an ICER of CAD 115,396 per QALY gained.

## 4. Discussion

In this study, we show that brexu-cel is a potentially cost-effective use of medical resources compared with best supportive care in Canada. Our analysis was conducted in line with the approved Health Canada indication for brexu-cel, and we used clinical data from the ZUMA-2 trial to populate the model and included comparators that were reflective of current Canadian clinical practice. In order to examine the plausibility of comparing the available meta-analysis results with the outcomes observed in ZUMA-2, various adjustments were explored to support the comparability of these two sources of evidence.

The published evidence has suggested that patients with DLBCL who achieve event-free survival at 24 months after frontline therapy have a subsequent OS similar to that of the age- and sex-matched general population [36]; however, this was not deemed transferable to a relapsed/refractory MCL population a priori. We therefore felt that 5 years of progression-free survival was a more realistic assumption for when patients could potentially achieve OS similar to the age- and sex-matched general population, as suggested in previous studies [59]. This further supports the validity of our results and helps to diminish the uncertainty inherent in parametric survival extrapolations.

In Canada, both the Institut national d’excellence en santé et services sociaux (INESSS), the HTA body responsible for the province of Quebec, and CADTH have published recommendations for public funding of brexu-cel, conditional upon carrying out additional clinical follow-up and mitigation of the economic burden. INESSS suggested a base case ICUR of between 151,390 CAD/QALY and 338,510 CAD/QALY gained [60], and CADTH was unable to determine a base case ICUR21. In the UK, NICE reported a base case ICUR of 46,898 GBP/QALY gained [20]. A US study from 2021 evaluated the cost-effectiveness of brexu-cel compared with best supportive care [61]. Using a US payer perspective, the authors concluded that brexu-cel was cost-effective with a base case ICER estimated to be 31,985 USD/QALY gained. While there is no explicit willingness-to-pay threshold in Canada, 100,000 CAD/QALY gained is commonly cited for oncology products [58]. A lower (50,000 CAD/QALY) threshold, which is often used for non-oncology products in Canada, or a higher (150,000 USD/QALY) threshold, which is used by I.C.E.R. in the United States, could be applied to our analysis. However, the 100,000 CAD/QALY gained threshold may be the most appropriate for comparison with previous oncology cost-effectiveness research in Canada [58]. The assessment details described above demonstrate that cost-effectiveness results often differ across regions and HTA agencies, due largely to differences in healthcare systems and reimbursement submission requirements and can lead to potentially divergent recommendations. These differences highlight the need for jurisdiction-specific estimates of cost-effectiveness. In addition, not all HTA agencies publish the full details of their assessments, underscoring the need for peer-reviewed analyses to be available in the public domain.

A key limitation of the economic analysis is the lack of randomized control trial evidence comparing brexu-cel against BSC. While a direct comparison of results between randomized treatment arms may minimize bias and is ideal for establishing comparative efficacy, given the lack of a recognized standard of care in the post-BTKi setting and the inherent problem of clinical equipoise in comparing CAR T to commonly used regimens known to have poor outcomes in this MCL patient population, a phase III trial may not be methodologically or ethically feasible. In addition, while this is a limitation common to most, if not all clinical trial data, the clinical environment of ZUMA-2 was highly controlled, and therefore, the results of the trial may not be representative of patient experience in the real-world setting. However, recent RWE studies of CAR T therapies in DLBCL have suggested that adverse events, response rates, and efficacy are similar in the real-world setting [32,62]. The current analysis also lacks EQ5D data from ZUMA-2. Despite this shortcoming, a commonly accepted alternative approach was followed in which the utility values were derived from the published oncology literature in MCL [56].

Another limitation is that the potential use of further therapy after relapse following CAR T cell administration was not included in model, as it is not clear how frequently such intervention occurs. Due to a lack of data, subsequent therapies received in the post-progression health state were not explicitly modeled. However, the impact of this assumption is very likely to be minimal, given the lack of effective therapies available in the pre-progression health state and the fact that the therapies available for post-progression treatment are likely to be similar between the two treatment arms. The results from a single-centre case series study from the US suggest that a small subset of patients may receive a range of subsequent salvage therapies following brexu-cel, which can include chemo-immunotherapy with/without local radiation, venetoclax, acalabrutinib, copanlisib, and abemaciclib [63].

A further limitation concerns the immaturity of the ZUMA-2 trial data used in this analysis. The model estimated long-term survival based on survival estimates with a median follow-up of at least 12 months, and while neither median PFS nor median OS was met by this time, the results are broadly in line with previous trials in CAR T [64,65], which supports the present analyses. However, as a majority of the time horizon in the model was based on extrapolated data, the associated uncertainty and results should be interpreted with caution.

The recent regulatory approval of brexu-cel in the US, EU, and Canada highlights the promising impact of this novel therapy for R/R MCL patients. While clinical and economic uncertainty are inherent in economic modeling exercises, the results of our base case analysis suggest that brexu-cel may be a cost-effective use of medical resources compared with best supportive care in Canada. However, additional research is required to confirm our results, as residual uncertainty around clinical outcomes for patients post-brexu-cel, illustrated in the special interest sensitivity analysis, warrant further assessment with longer follow-up data. This will inform even more robust future cost-effectiveness analyses. Future studies could also examine the frequency with which relapsing patients receive available subsequent therapies to further build on these findings.

## Figures and Tables

**Table 1:** Summary of modeling approach.

Elements	Description
Target population	ZUMA-2 trial population (R/R mantle cell lymphoma following treatment with a BTKi
Treatments	Brexucabtagene autoleucel vs. BSC
Model design	Partitioned survival mixture cure model for brexucabtagene autoleucel Partitioned survival model for BSC
Model inputs	Efficacy (PFS and OS), safety Utility values Treatment-related costs, disease-related costs, end-of-life costs
Outcomes of interest	Costs by category LYs and QALYs Incremental costs, incremental LYs, incremental QALYs Incremental cost/LY and cost/QALY gained
Perspective	Canadian healthcare system perspective
Health states	Pre-progression survival Post-progression survival Death
Time horizon	Canadian healthcare system perspective
Discount	1.5% per year for both costs and outcomes
Cycle length	1 month
Year of cost and currency	2021 Canadian dollar
Sensitivity analysis	One-way deterministic sensitivity analyses Probabilistic sensitivity analyses Scenario analyses
Programming software	Microsoft Excel 365

Abbreviations: BSC, Best supportive care; BTKi, Bruton’s tyrosine kinase inhibitor; LYs, Life-years; OS, overall survival; PFS, progression-free survival; QALY, quality-adjusted life-year; R/R, relapsed or refractory.

**Table 2:** Proportion of patients on each of BSC treatments.

Treatment	Proportion of Patients on Intervention in Base Case (%)
Rituximab	68.2%
Bendamustine	57.4%
Bortezomib	5.5%
Anthracycline-based	7.3%
Total	138.5%

Note: Sum is more than 100% as patients can be given these drugs in combination.

**Table 3:** Estimated utility values from literature used in model base case.

Health States	Value	Standard Error	Reference
Pre-progression	0.780	0.010	NICE ibrutinib, 2016 [56]
Pre-progression for long-term survivors	0.812	0.010	Calculated from Ara and Brazier, 2010 [57]
Post-progression	0.680	0.024	NICE ibrutinib, 2016 [56]

Abbreviation: NICE: National Institute for Health and Clinical Excellence.

**Table 4:** Disaggregated deterministic results of brexucabtagene autoleucel vs. BSC.

	Brexucabtagene Autoleucel	Literature-Based Meta-Analysis	Incremental
Median survival (years)	12.71	0.88	11.83
Total undiscounted years	13.22	1.76	11.46
Pre-progression	9.30	1.68	7.63
Post-progression	3.92	0.09	3.83
Total discounted years	11.26	1.70	9.56
Pre-progression	7.95	1.63	6.33
Post-progression	3.31	0.08	3.23
Total discounted QALYs	8.34	1.31	7.03
Pre-progression	6.23	1.27	4.97
Pre-Progression, pre-cure point	1.95	1.11	0.85
Pre-Progression, post-cure point	4.28	0.16	4.12
Post-progression	2.14	0.05	2.09
Adverse events	−0.04	−0.01	−0.03
Total discounted costs	CAD 688,040	CAD 66,108	CAD 621,933
Total treatment-related costs	CAD 589,375	CAD 27,946	CAD 561,429
Total drug acquisition	CAD 533,523	CAD 27,221	CAD 506,302
Total apheresis	CAD 1392	CAD 0	CAD 1392
Total drug administration	CAD 211	CAD 726	CAD −515
Total lymphodepletion chemotherapy	CAD 646	CAD 0	CAD 646
Total bridging therapy	CAD 220	CAD 0	CAD 220
Total hospitalization	CAD 53,383	CAD 0	CAD 3383
Total disease management	CAD 57,739	CAD 2490	CAD 55,249
Pre-progression	CAD 3939	CAD 1225	CAD 2714
Post-progression	CAD 53,800	CAD 1264	CAD 52,535
Other costs	CAD 40,926	CAD 35,671	CAD 5255
End of life care	CAD 29,582	CAD 34,589	CAD −5007
Adverse events	CAD 11,344	CAD 1082	CAD 10,262
		Cost/QALY	CAD 88,503

Abbreviations: BSC, best supportive care; QALYs: quality-adjusted life-years.

**Figure A1:**
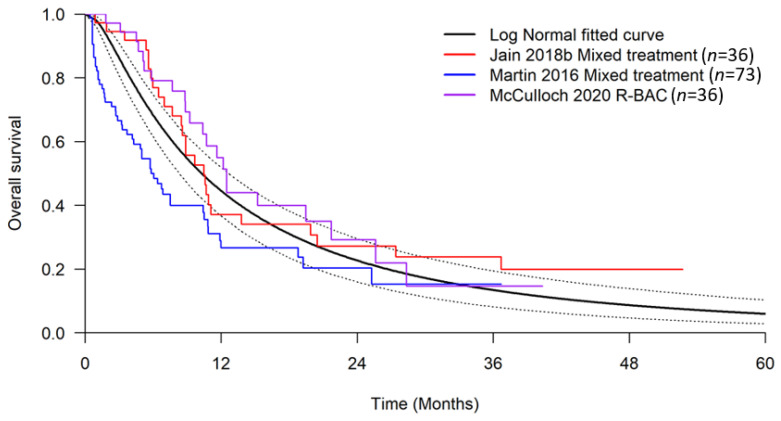
Random effects meta-analysis (log-normal model) of overall survival Kaplan–Meier curves; studies with mixed treatments or R-BAC. Abbreviations: R-BAC = Rituximab-Bendamustine Cytarabine.

**Figure A2:**
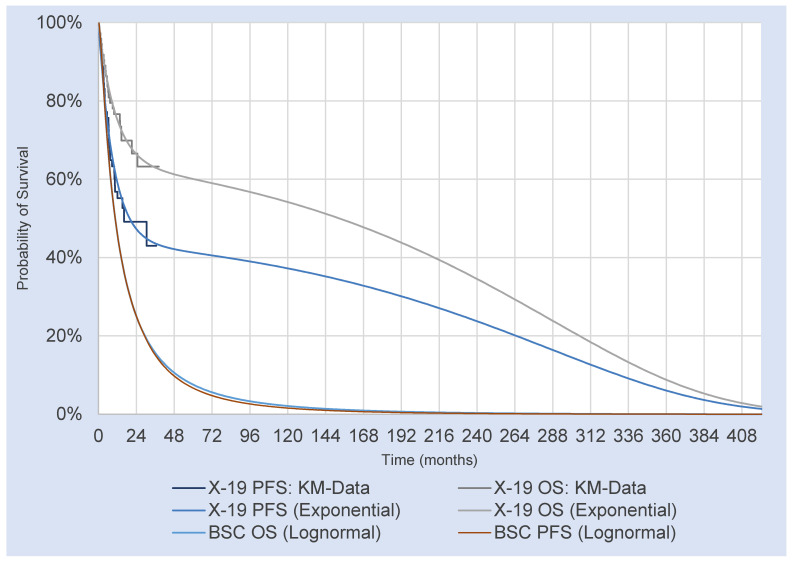
Mixture cure parametric model extrapolations fitted to overall survival and progression-free survival data of brexucabtagene autoleucel (data from ZUMA-2 trial) and BSC (data from meta-analysis). Data derived from ZUMA-2 trial (Brexu-cel) and meta-analysis (BSC). Abbreviations: PFS: progression-free survival; OS: overall survival; ITT: intent-to-treat population; KM: Kaplan–Meier; PS-MCM: partitioned survival mixed cure model.

## Data Availability

Data are contained within the article and Appendix A.

## Appendix A

**Table A1:** Summary of health state resource use frequency, based on Canadian Clinical Expert feedback.

Resource		Progression-Free		Progression-Free Post-5 Years		Progressed	
		% of Patients	Frequency per Cycle	% of Patients	Frequency per Cycle	% of Patients	Frequency per Cycle
Physician visits	Specialist visit	100%	0.33	100%	0.17	100%	1.00
Laboratory tests	Complete Blood Count	100%	0.33	0%	0.00	100%	1.00
	Lactate Dehydrogenase	100%	0.33	0%	0.00	100%	1.00
	Blood glucose	100%	0.33	0%	0.00	100%	1.00
Radiology	CT scan	100%	0.33	0%	0.00	100%	0.33
	X-ray	100%	0.17	0%	0.00	100%	0.33
Hospitalization		0%	0.00	0%	0.00	100%	0.08

Abbreviations: CT, computed tomography.

**Table A2:** Extended cost table, including unit cost per medical resource.

Resource	Unit Cost	Reference
Specialist visit	CAD 157.00	Ontario MoHLTC Schedule of Benefits—Physician Services, Haematology Consultation [43]
Complete blood count	CAD 3.98	Ontario MoHLTC Schedule of Benefits—Laboratory Services, Hematology—CBC [55]
Lactate dehydrogenase	CAD 1.28	Ontario MoHLTC Schedule of Benefits—Laboratory Services, Lactate Dehydrogenase [55]
Blood glucose	CAD 1.28	Ontario MoHLTC Schedule of Benefits—Laboratory Services, C-reactive Protein [55]
CT scan (abdominal, thorax)	CAD 195.00	Ontario MoHLTC Schedule of Benefits—Physician Services, CT abdomen and thorax, with and without IV contrast. [43]
X-ray	CAD 32.25	Ontario MoHLTC Schedule of Benefits—Physician Services, Skeletal survey studies; assumed 3 views. [43]
Hospitalization	CAD 12,756.57	CIHI Patient Cost Estimator [45]
IV administration	CAD 54.25	Ontario Schedule of Benefits for Physician Services. [43]
Conditional chemotherapy administration	CAD 105.15	Ontario Schedule of Benefits for Physician Services. [43]
Palliative care (one-off)	CAD 34,037	Walker et al. 2011. [41]
Office visit	CAD 157.00	Ontario Schedule of Benefits for Physician Services. [43]
Brexucabtagene autoleucel one-time treatment cost	CAD 533,523.10	Kite list price
Brexucabtagene autoleucel administration	CAD 185.00	Ontario Schedule of Benefits for Physician Services. [43]
Apheresis	CAD 1343.98	Holbro et al. 2013. [44].
Adverse event: cytokine release syndrome	CAD 18,366.96	Cost of 6 days of tocilizumab treatment and 11 days hospitalized. Fifty-nine percent of patients were treated with tocilizumab. Cost per hospital day is weighted average of cost per inpatient day from CIHI patient cost estimator. [45]

Abbreviations: CIHI, Canadian Institute for Health Information; CT, computed tomography; MoHLTC, Ministry of Health and Long-Term Care.

**Table A3:** Unit drug costs.

Resource	Unit Cost	Reference
Rituximab 100 mg	CAD 482.31	Ontario Exceptional Access Program [47]
Bendamustine 25 mg	CAD 312.50	pCODR Economic Guidance Report for Bendamustine [48]
Lenalidomide 25 mg	CAD 424.00	Ontario Exceptional Access Program [47]
Lenalidomide 20 mg	CAD 403.00	Ontario Exceptional Access Program [47]
Lenalidomide 15 mg	CAD 382.00	Ontario Exceptional Access Program [47]
Lenalidomide 10 mg	CAD 361.00	Ontario Exceptional Access Program [47]
Lenalidomide 5 mg	CAD 340.00	Ontario Exceptional Access Program [47]
Lenalidomide 2.5 mg	CAD 329.00	Ontario Exceptional Access Program [47]
Bortezomib 3.5 mg	CAD 1402.42	pCODR Economic Guidance Report for Daratumumab [49]
Anthracycline 1 mg	CAD 5.05	pCODR Economic Guidance Report for Pertuzumab-Trastuzumab [50]
Fludarabine 50 mg	CAD 255.00	pCODR Economic Guidance Report for Ibrutinib [5]
Cyclophosphamide 1g	CAD 52.06	pCODR Economic Guidance Report for Ibrutinib [5]
Ibrutinib 140 mg	CAD 97.60	Ontario Exceptional Access Program [47]
Dexamethasone 4 mg	CAD 0.30	Ontario Drug Benefit Formulary [66]
Tocilizumab 20 mg	CAD 182.80	Ontario Exceptional Access Program [47]

Abbreviations: MoHLTC, Ministry of Health and Long-Term Care; pCODR, pan-Canadian Oncology Drug Review.

**Table A4:** Dosing of best supportive care therapies.

Chemotherapy	Admin Route	mg/m^2^/day	Frequency	mg/Unit	Cost/Unit	Source
Rituximab	IV	375	Q4W for 6 cycles [54]	100	482.31	Ontario EAP [47]
Bendamustine	IV	70	Q4W 2 days for 6 cycles [54]	25	312.50	pCODR economic review of Bendamustine [48]
Lenalidomide	Oral	25	21 days on, 7 off [53]	25	424.00	Ontario EAP [47]
Bortezomib	IV	1.3	Q3W 4 days, 9 cycles [51]	3.5	1402.42	pCODR economic review of Daratumumab [49]
Anthracycline	IV	50	Q3W [52]	1	5.05	pCODR economic review of Pertuzumab-Trastuzumab [50]

Abbreviations: BSC, best supportive care; EAP, Exceptional Access Program; IV, intravenous; pCODR, pan-Canadian Oncology Drug Review; Q3W, every 3 weeks; Q4W, every 4 weeks.

**Table A5:** Hospitalization of Patients.

Item	Value	S.E.	Source
Proportion of brexu-cel patients who visit ICU	22.7%		ZUMA-2 Clinical Study Report [38]
% of BSC patients who visit ICU	0	0	~
Average duration in ICU	21.2 days	1.80	ZUMA-2 Clinical Study report [38]
Total hospitalization cost	CAD 63,758.81	0	~

Abbreviation: BSC, best supportive care.

**Table A6:** Adverse event rates from ZUMA-2.

	Incidence	
Adverse Events	Brexucabtagene Autoleucel (%) (se)	BSC (%) (se)
Cytokine release syndrome (CRS) Grade ≥2	62 (6)	0 (0)
Pyrexia	13 (4)	0 (0)
Anemia	50 (6)	0 (0)
Platelet count decreased	38 (6)	0 (0)
Hypotension	22 (5)	0 (0)
Neutrophil count decreased	50 (6)	0 (0)
White blood cell count decreased	40 (6)	0 (0)
Hypoxia	21 (5)	0 (0)
Hypophosphatemia	22 (5)	0 (0)
Neutropenia	34 (6)	0 (0)
Hyponatremia	10 (4)	0 (0)
ALT increased	9 (3)	0 (0)
Encephalopathy	19 (5)	0 (0)
Hypokalemia	7 (3)	0 (0)
Hypocalcemia	6 (3)	0 (0)
Thrombocytopenia	16 (4)	0 (0)
AST increased	10 (4)	0 (0)
Confusional state	12 (4)	0 (0)
Hyperglycemia	6 (3)	0 (0)
Hypertension	13 (4)	0 (0)
Acute Kidney Injury	7 (3)	0 (0)
Leukopenia	13 (4)	0 (0)
Lymphocyte count decreased	9 (3)	0 (0)
Pneumonia	9 (3)	0 (0)
Respiratory Failure	6 (3)	0 (0)
Sepsis	6 (3)	0 (0)

Abbreviations: ALT: alanine aminotransferase; AST aspartate aminotransferase; BSC, best supportive care; SE, standard error.

**Table A7:** Disaggregated cost and outcomes of brexucabtagene autoleucel vs. BSC based on 1000 iterations.

	Brexucabtagene Autoleucel	BSC	Incremental
Total discounted years	11.21	1.72	9.49
Pre-progression	8.01	1.50	6.51
Post-progression	3.20	0.22	2.98
Total discounted QALYs	8.31	1.31	7.00
Pre-progression	6.28	1.17	5.11
Pre-progression, pre-cure point	1.99	1.03	0.95
Pre-progression, post-cure point	4.29	0.14	4.15
Post-progression	2.07	0.14	1.92
Adverse events	−0.04	−0.01	−0.03
Total discounted costs	CAD 689,636	CAD 68,066	CAD 621,571
Total treatment-related costs	CAD 592,182	CAD 27,701	CAD 564,480
Total drug acquisition	CAD 533,523	CAD 26,989	CAD 506,534
Total apheresis	CAD 1374	CAD 0	CAD 1374
Total drug administration	CAD 211	CAD 713	CAD −502
Total lymphodepletion chemotherapy	CAD 646	CAD 0	CAD 646
Total bridging therapy	CAD 222	CAD 0	CAD 222
Total hospitalization	CAD 56,206	CAD 0	CAD 56,206
Total disease management	CAD 56,500	CAD 4692	CAD 51,808
Pre-progression	CAD 3996	CAD 1145	CAD 2851
Post-progression	CAD 52,504	CAD 3547	CAD 48,957
Other costs	CAD 40,954	CAD 35,672	CAD 5282
End-of-life care	CAD 29,603	CAD 34,591	CAD −4988
Adverse events	CAD 11,351	CAD 1081	CAD 10,270
		Cost/QALY	CAD 88,814

Abbreviations: BSC, best supportive care; LYs, life-years; QALYs, quality-adjusted life-years.

**Table A8:** Parameter values varied in deterministic sensitivity analyses.

Parameter	Original Value	Lower Limit	Upper Limit
Patient Characteristics			
Bodyweight	81.8000	77.9723	85.6277
BSA	1.9780	1.9251	2.0309
Resource Use			
Pre-Progression Resource Use: Full blood count	0.3333	0.2157	0.4761
Pre-Progression Resource Use: X-ray	0.1667	0.1079	0.2381
Pre-Progression Resource Use: Blood glucose	0.3333	0.2157	0.4761
Pre-Progression Resource Use: Lactate dehydrogenase	0.3333	0.2157	0.4761
Pre-Progression Resource Use: CT Scan	0.1667	0.1079	0.2381
Pre-Progression Resource Use: Office visit	0.1667	0.1079	0.2381
Pre-Progression Cured: Resource Use: Office visit	0.1667	0.1079	0.2381
Post-Progression Resource Use: Full blood count	1.0000	0.6471	1.4284
Post-Progression Resource Use: X-ray	0.3333	0.2157	0.4761
Post-Progression Resource Use: Blood glucose	1.0000	0.6471	1.4284
Post-Progression Resource Use: Lactate dehydrogenase	1.0000	0.6471	1.4284
Post-Progression Resource Use: Office visit	1.0000	0.6471	1.4284
Post-Progression Resource Use: CT Scan	0.3333	0.2157	0.4761
Post-Progression Resource Use: Hospitalization	0.0833	0.0539	0.1190
End-of-life Resource Use: Palliative care (one-off)	1.0000	0.6471	1.4284
Brexucabtagene autoleucel—Proportion ICU Stay	0.2200	0.1402	0.3119
Brexucabtagene autoleucel—Proportion Non-ICU—Hospital days	16.5000	−4.5696	37.5696
Bridging Therapy Proportion	0.3676	0.3133	0.4237
Cost: Initial Hospitalization: Intensive Care Unit Day	CAD 8,343.7300	CAD 5399.6221	1 CAD 1918.2165
Cost: Initial Hospitalization: Inpatient Day (Non-ICU)	CAD 1580.5300	CAD 1022.8357	CAD 2257.6352
Cost: Stem cell transplant	CAD 166,855.5300	CAD 10,7980.1014	CAD 23,8337.0904
Cost: Office visit	CAD 174.6400	CAD 113.0178	CAD 249.4565
Cost: Palliative care (one-off)	CAD 35,262.4800	CAD 22,820.0178	CAD 50,369.0641
Cost: Full blood count	CAD 4.1200	CAD 2.6662	CAD 5.8850
Cost: X-ray	CAD 23.1500	CAD 14.9815	CAD 33.0676
Cost: Blood glucose	CAD 1.3300	CAD 0.8607	CAD 1.8998
Cost: Lactate dehydrogenase	CAD 1.3300	CAD 0.8607	CAD 1.8998
Cost: Inpatient stay	CAD 1580.5300	CAD 1022.8357	CAD 2257.6352
Cost: CT Scan	CAD 195.0000	CAD 126.1937	CAD 278.5388
Cost: Hospitalization	CAD 13,215.8400	CAD 8552.5948	CAD 18,877.5574
Utility			
Utility: Pre-progression (up to 60 months)	0.7800	0.7601	0.7993
Utility: Pre-progression, cured (beyond 60 months)	0.7852	0.7653	0.8045
Utility: Post-progression	0.6800	0.6321	0.7261
Adverse Events			
Brexucabtagene autoleucel AE incidence: Hypotension	0.2206	0.1305	0.3265
Brexucabtagene autoleucel AE incidence: Neutrophil count decreased	0.5294	0.4103	0.6468
Brexucabtagene autoleucel AE incidence: White blood cell count decreased	0.4118	0.2977	0.5308
Brexucabtagene autoleucel AE incidence: Hypoxia	0.2059	0.1186	0.3097
Brexucabtagene autoleucel AE incidence: Hypophosphataemia	0.2206	0.1305	0.3265
Brexucabtagene autoleucel AE incidence: Neutropenia	0.3382	0.2308	0.4548
Brexucabtagene autoleucel AE incidence: Hyponatraemia	0.1029	0.0427	0.1855
Brexucabtagene autoleucel AE incidence: Alanine aminotransferase increased	0.0882	0.0333	0.1663
Brexucabtagene autoleucel AE incidence: Encephalopathy	0.1765	0.0956	0.2756
Brexucabtagene autoleucel AE incidence: Hypokalaemia	0.1471	0.0735	0.2405
Brexucabtagene autoleucel AE incidence: Hypocalcaemia	0.0882	0.0333	0.1663
Brexucabtagene autoleucel AE incidence: Thrombocytopenia	0.1618	0.0844	0.2582
Brexucabtagene autoleucel AE incidence: Aspartate aminotransferase increased	0.1029	0.0427	0.1855
Brexucabtagene autoleucel AE incidence: Confusional state	0.1176	0.0526	0.2042
Brexucabtagene autoleucel AE incidence: Hypertension	0.1324	0.0629	0.2225
Brexucabtagene autoleucel AE incidence: Acute Kidney Injury	0.0735	0.0244	0.1465
Brexucabtagene autoleucel AE incidence: Leukopenia	0.1471	0.0735	0.2405
Brexucabtagene autoleucel AE incidence: Lymphocyte count decreased	0.0882	0.0333	0.1663
Brexucabtagene autoleucel AE incidence: Pneumonia	0.1324	0.0629	0.2225
Brexucabtagene autoleucel AE incidence: Respiratory Failure	0.0588	0.0163	0.1259
Brexucabtagene autoleucel AE incidence: Sepsis	0.0588	0.0163	0.1259
Disutility Cytokine release syndrome	0.7800	0.4087	0.9850
Disutility Pyrexia	0.1100	0.0707	0.1566
Disutility Anaemia	0.1200	0.0771	0.1708
Disutility Platelet Count decreased	0.1100	0.0707	0.1566
Disutility Hypotension	0.1500	0.0961	0.2133
Disutility Neutrophil count decreased	0.1500	0.0961	0.2133
Disutility White blood cell count decreased	0.1500	0.0961	0.2133
Disutility Hypoxia	0.1100	0.0707	0.1566
Disutility Hypophosphataemia	0.1500	0.0961	0.2133
Disutility Neutropenia	0.0900	0.0579	0.1282
Disutility Hyponatraemia	0.1500	0.0961	0.2133
Disutility Alanine aminotransferase increased	0.1500	0.0961	0.2133
Disutility Encephalopathy	0.1500	0.0961	0.2133
Disutility Hypokalaemia	0.1500	0.0961	0.2133
Disutility Hypocalcaemia	0.1500	0.0961	0.2133
Disutility Thrombocytopenia	0.1100	0.0707	0.1566
Disutility Aspartate aminotransferase increased	0.1500	0.0961	0.2133
Disutility Confusional state	0.1500	0.0961	0.2133
Disutility Hypertension	0.1500	0.0961	0.2133
Disutility Acute Kidney Injury	0.1500	0.0961	0.2133
Disutility Leukopenia	0.1500	0.0961	0.2133
Disutility Lymphocyte count decreased	0.1500	0.0961	0.2133
Disutility Pneumonia	0.1500	0.0961	0.2133
Disutility Respiratory Failure	0.1500	0.0961	0.2133
Disutility Sepsis	0.1500	0.0961	0.2133
Duration Cytokine release syndrome	4.0000	2.5886	5.7136
Duration Pyrexia	2.0000	1.2943	2.8568
Duration Anaemia	14.0000	9.0601	19.9977
Duration Platelet Count decreased	50.0000	32.3574	71.4202
Duration Hypotension	5.0000	3.2357	7.1420
Duration Neutrophil count decreased	17.0000	11.0015	24.2829
Duration White blood cell count decreased	40.0000	25.8859	57.1362
Duration Hypoxia	2.0000	1.2943	2.8568
Duration Hypophosphataemia	5.0000	3.2357	7.1420
Duration Neutropenia	47.0000	30.4159	67.1350
Duration Hyponatraemia	7.0000	4.5300	9.9988
Duration Alanine aminotransferase increased	7.0000	4.5300	9.9988
Duration Encephalopathy	9.0000	5.8243	12.8556
Duration Hypokalaemia	7.0000	4.5300	9.9988
Duration Hypocalcaemia	7.0000	4.5300	9.9988
Duration Thrombocytopenia	63.0000	40.7703	89.9894
Duration Aspartate aminotransferase increased	7.0000	4.5300	9.9988
Duration Confusional state	7.0000	4.5300	9.9988
Duration Hypertension	5.0000	3.2357	7.1420
Duration Acute Kidney Injury	7.0000	4.5300	9.9988
Duration Leukopenia	21.0000	13.5901	29.9965
Duration Lymphocyte count decreased	64.0000	41.4174	91.4178
Duration Pneumonia	7.0000	4.5300	9.9988
Duration Respiratory Failure	7.0000	4.5300	9.9988
Duration Sepsis	7.0000	4.5300	9.9988
AE cost: Cytokine release syndrome	CAD 18,366.9647	CAD 11,886.1311	CAD 26,235.4440

Abbreviations: BSA = body surface area; AE = adverse events; ICU intensive care unit.

**Table A9:** Parameter values varied in probabilistic sensitivity analyses.

Parameter	Value	SE	Distribution
Patient Characteristics			
Bodyweight	81.8 kg	1.95296	Normal
BSA	1.978 m^2^	0.026997	Normal
Resource Use			
Pre-Progression Resource Use: Full blood count	0.33	0.066667	Gamma
Pre-Progression Resource Use: X-ray	0.17	0.033333	Gamma
Pre-Progression Resource Use: Blood glucose	0.33	0.066667	Gamma
Pre-Progression Resource Use: Lactate dehydrogenase	0.33	0.066667	Gamma
Pre-Progression Resource Use: CT Scan	0.17	0.033333	Gamma
Pre-Progression Resource Use: Office visit	0.17	0.033333	Gamma
Post-Progression Resource Use: Full blood count	1.00	0.2	Gamma
Post-Progression Resource Use: X-ray	0.33	0.066667	Gamma
Post-Progression Resource Use: Blood glucose	1.00	0.2	Gamma
Post-Progression Resource Use: Lactate dehydrogenase	1.00	0.2	Gamma
Post-Progression Resource Use: Office visit	1.00	0.2	Gamma
Post-Progression Resource Use: CT Scan	0.33	0.066667	Gamma
Post-Progression Resource Use: Hospitalization	0.08	0.016667	Gamma
End-of-life Palliative care (one-off)	1	0.20	Gamma
Apheresis: One-Time cost	CAD 1392.37	CAD 278.47	Gamma
Tecartus Proportion ICU Stay	0.22	0.04	Beta
Hospital days, proportion ICU	18	22.5	Normal
Hospital days, proportion non-ICU	16.50	10.75	Normal
Proportion requiring bridging therapy	0.37	0.03	Beta
Cost: Initial Hospitalization: Intensive Care Unit Day	CAD 8343.73	CAD 1668.75	Gamma
Cost: Initial Hospitalization: Inpatient Day (Non-ICU)	CAD 1580.53	CAD 316.11	Gamma
Cost: Stem cell transplant	CAD 166,855.53	CAD 33,371.11	Gamma
Cost: Office visit	CAD 174.64	CAD 34.93	Gamma
Cost: Palliative care (one-off)	CAD 35,262.48	CAD 7052.50	Gamma
Cost: Full blood count	CAD 4.12	CAD 0.82	Gamma
Cost: X-ray	CAD 23.15	CAD 4.63	Gamma
Cost: Blood glucose	CAD 1.33	CAD 0.27	Gamma
Cost: Lactate dehydrogenase	CAD 1.33	CAD 0.27	Gamma
Cost: Inpatient stay	CAD 1580.53	CAD 316.11	Gamma
Cost: CT Scan	CAD 195.00	CAD 39.00	Gamma
Cost: Hospitalization	CAD 13,215.84	CAD 2643.17	Gamma
Utility			
Utility: Pre-progression (up to 60 months)	0.78	0.01	Beta
Utility: Pre-progression, cured (beyond 60 months)	0.7851841	0.01	Beta
Utility: Post-progression	0.68	0.024	Beta
Adverse Events			
Brexucabtagene autoleucel AE incidence: Cytokine release syndrome (CRS)	62%	0.059	Beta
Brexucabtagene autoleucel AE incidence: Pyrexia	15%	0.043	Beta
Brexucabtagene autoleucel AE incidence: Anaemia	51%	0.061	Beta
Brexucabtagene autoleucel AE incidence: Platelet Count decreased	38%	0.059	Beta
Brexucabtagene autoleucel AE incidence: Hypotension	22%	0.050	Beta
Brexucabtagene autoleucel AE incidence: Neutrophil count decreased	53%	0.061	Beta
Brexucabtagene autoleucel AE incidence: White blood cell count decreased	41%	0.060	Beta
Brexucabtagene autoleucel AE incidence: Hypoxia	21%	0.049	Beta
Brexucabtagene autoleucel AE incidence: Hypophosphataemia	22%	0.050	Beta
Brexucabtagene autoleucel AE incidence: Neutropenia	34%	0.057	Beta
Brexucabtagene autoleucel AE incidence: Hyponatraemia	10%	0.037	Beta
Brexucabtagene autoleucel AE incidence: Alanine aminotransferase increased	9%	0.034	Beta
Brexucabtagene autoleucel AE incidence: Encephalopathy	18%	0.046	Beta
Brexucabtagene autoleucel AE incidence: Hypokalaemia	15%	0.043	Beta
Brexucabtagene autoleucel AE incidence: Hypocalcaemia	9%	0.034	Beta
Brexucabtagene autoleucel AE incidence: Thrombocytopenia	16%	0.045	Beta
Brexucabtagene autoleucel AE incidence: Aspartate aminotransferase increased	10%	0.037	Beta
Brexucabtagene autoleucel AE incidence: Confusional state	12%	0.039	Beta
Brexucabtagene autoleucel AE incidence: Hypertension	13%	0.041	Beta
Brexucabtagene autoleucel AE incidence: Acute Kidney Injury	7%	0.032	Beta
Brexucabtagene autoleucel AE incidence: Leukopenia	15%	0.043	Beta
Brexucabtagene autoleucel AE incidence: Lymphocyte count decreased	9%	0.034	Beta
Brexucabtagene autoleucel AE incidence: Pneumonia	13%	0.041	Beta
Brexucabtagene autoleucel AE incidence: Respiratory Failure	6%	0.029	Beta
Brexucabtagene autoleucel AE incidence: Sepsis	6%	0.029	Beta
Disutility Cytokine release syndrome	0.78	0.156	Beta
Disutility Pyrexia	0.11	0.022	Beta
Disutility Anaemia	0.12	0.024	Beta
Disutility Platelet Count decreased	0.11	0.022	Beta
Disutility Hypotension	0.15	0.03	Beta
Disutility Neutrophil count decreased	0.15	0.03	Beta
Disutility White blood cell count decreased	0.15	0.03	Beta
Disutility Hypoxia	0.11	0.022	Beta
Disutility Hypophosphataemia	0.15	0.03	Beta
Disutility Neutropenia	0.09	0.018	Beta
Disutility Hyponatraemia	0.15	0.03	Beta
Disutility Alanine aminotransferase increased	0.15	0.03	Beta
Disutility Encephalopathy	0.15	0.03	Beta
Disutility Hypokalaemia	0.15	0.03	Beta
Disutility Hypocalcaemia	0.15	0.03	Beta
Disutility Thrombocytopenia	0.11	0.022	Beta
Disutility Aspartate aminotransferase increased	0.15	0.03	Beta
Disutility Confusional state	0.15	0.03	Beta
Disutility Hypertension	0.15	0.03	Beta
Disutility Acute Kidney Injury	0.15	0.03	Beta
Disutility Leukopenia	0.15	0.03	Beta
Disutility Lymphocyte count decreased	0.15	0.03	Beta
Disutility Pneumonia	0.15	0.03	Beta
Disutility Respiratory Failure	0.15	0.03	Beta
Disutility Sepsis	0.15	0.03	Beta
Duration Cytokine release syndrome	4	0.8	Gamma
Duration Pyrexia	2	0.4	Gamma
Duration Anaemia	14	2.8	Gamma
Duration Platelet Count decreased	50	10	Gamma
Duration Hypotension	5	1	Gamma
Duration Neutrophil count decreased	17	3.4	Gamma
Duration White blood cell count decreased	40	8	Gamma
Duration Hypoxia	2	0.4	Gamma
Duration Hypophosphataemia	5	1	Gamma
Duration Neutropenia	47	9.4	Gamma
Duration Hyponatraemia	7	1.4	Gamma
Duration Alanine aminotransferase increased	7	1.4	Gamma
Duration Encephalopathy	9	1.8	Gamma
Duration Hypokalaemia	7	1.4	Gamma
Duration Hypocalcaemia	7	1.4	Gamma
Duration Thrombocytopenia	63	12.6	Gamma
Duration Aspartate aminotransferase increased	7	1.4	Gamma
Duration Confusional state	7	1.4	Gamma
Duration Hypertension	5	1	Gamma
Duration Acute Kidney Injury	7	1.4	Gamma
Duration Leukopenia	21	4.2	Gamma
Duration Lymphocyte count decreased	64	12.8	Gamma
Duration Pneumonia	7	1.4	Gamma
Duration Respiratory Failure	7	1.4	Gamma
Duration Sepsis	7	1.4	Gamma
AE cost: Cytokine release syndrome	CAD 18,366.96	CAD 3673.39	Gamma

Abbreviations: BSA = body surface area; AE = adverse events; ICU intensive care unit.
